# Stage‐Specific Patterns of Imaging and Non‐Motor Biomarkers in Spinocerebellar Ataxia Type 3

**DOI:** 10.1002/acn3.70555

**Published:** 2026-10-13

**Authors:** Chun Li, Zhi‐Xian Ye, Xuan‐Yu Chen, Liang‐Liang Qiu, Zhi‐li Chen, Yu‐Sen Qiu, Wei Lin, Ru‐Ying Yuan, Xin‐Tong Yu, Chun‐Yu Huang, Xin‐Yuan Chen, Min‐Ting Lin, Jian‐Ping Hu, Wan‐Jin Chen, Ning Wang, Shao‐Bo Yao, Shi‐Rui Gan, Ying Fu

**Affiliations:** ^1^ Department of Neurology and Institute of Neurology of First Affiliated Hospital Institute of Neuroscience, and Fujian Key Laboratory of Molecular Neurology, Fujian Medical University Fuzhou China; ^2^ Department of Neurology National Regional Medical Center, Binhai Campus of the First Affiliated Hospital, Fujian Medical University Fuzhou China; ^3^ Department of Neurology, Xinqiao Hospital and the Second Affiliated Hospital Army Medical University (Third Military Medical University) Chongqing China; ^4^ Department of Rehabilitation Medicine of First Affiliated Hospital Fujian Medical University Fuzhou China; ^5^ Department of Radiology of First Affiliated Hospital Fujian Medical University Fuzhou China

**Keywords:** biomarkers, MRI, spinocerebellar ataxia Type 3, TSPO‐PET

## Abstract

**Objective:**

Advancements in disease‐modifying therapies for spinocerebellar ataxia Type 3 (SCA3) underscore the need for robust biomarkers to monitor disease progression and evaluate treatment efficacy. Although motor phenotypes have been well characterized, less is known about non‐motor and imaging‐based features across different disease stages. This study aimed to characterize stage‐specific patterns of non‐motor and imaging biomarkers in SCA3.

**Methods:**

This cross‐observational study included 229 patients with SCA3 and 104 age‐ and sex‐matched healthy controls from the Organization for Southeast China for cerebellar ataxia research. Non‐motor symptoms were assessed using a battery of neuropsychological tests. Brain volume loss was assessed by 3.0‐Tesla MRI with regions‐of‐interest (ROI)‐based volumetric analysis. Neuroinflammation was quantified in vivo using translocator protein positron emission tomography (TSPO‐PET) with the ^18^F‐DPA‐714 radioligand. Additive regression models were fitted to evaluate the cross‐sectional associations between biomarkers and disease stages. Receiver operating curve (ROC) analyses were applied to estimate the discriminative ability of the candidate biomarkers.

**Results:**

Non‐motor symptoms, including cognitive functions, anxiety, fatigue, depression, and sleep quality, declined after ataxia onset in SCA3. Volume loss in the brainstem and ventral diencephalon diverged from normal controls at 0.7 and 0.2 years before ataxia onset, respectively. Correspondingly, the TSPO signal in the brainstem and ventral diencephalon increased and deviated from normal throughout the disease course.

**Interpretation:**

Our findings propose a staging model of SCA3 that includes a biomarker stage characterized by multidimensional objective indicators of neurodegeneration and neuroinflammation from pre‐ataxia to ataxia onset.

**Trial Registration:**

ClinicalTrials.gov identifier: NCT04010214

## 1 Introduction

Spinocerebellar ataxia Type 3 (SCA3), also known as Machado‐Joseph disease (MJD), is the most common form of spinocerebellar ataxia worldwide. It is caused by unstable expansions of polyglutamine encoding CAG repeats in the *ATXN*3 gene [1]. The pathophysiological characteristic of SCA3 is abnormally elongated ataxin‐3 protein aggregates in the nuclei, which results in the neurotoxic degeneration of neurons and neural networks in the cerebellum, brainstem, and other brain regions [2]. The onset of the disease typically occurs during adulthood, and the clinical manifestations are highly heterogeneous, including progressive ataxia, pyramidal signs, parkinsonism, and a range of non‐motor symptoms [3, 4]. These progressive manifestations significantly impair quality of life and contribute to premature death [5].

Currently, therapeutic strategies for SCA3 primarily focus on symptom management, with no available causal treatment. However, targeted therapies such as antisense oligonucleotides to downregulate the *ATXN3* gene are now in clinical trials (e.g., ClinicalTrials.gov: NCT05822908). Like other neurodegenerative diseases with a presymptomatic phase, there is an urgent need for reliable biomarkers to track the underlying pathophysiological evolution and inform the design of future preventive clinical trials in SCA3.

Several clinical and imaging biomarkers have been proposed for tracking SCA3 progression. The Scale for the Assessment and Rating of Ataxia (SARA) remains the most widely used clinical tool for evaluating motor impairment [6]. Neurofilament light chain (NfL), a marker of neuroaxonal damage, is elevated in the preataxic stage and correlates with motor symptoms severity [7, 8]. Structural MRI has revealed early volume loss in the cerebellum, brainstem, and basal ganglia, with progressive worsening across disease stages [9, 10]. While these findings establish the potential of biomarkers to detect early neurodegenerative changes, they do not fully capture the broader phenotypic spectrum of SCA3, particularly the pattern of non‐motor and neuroinflammatory features across disease stages.

Accumulated evidence has indicated that non‐motor symptoms such as cognitive impairment, depression, anxiety, and sleep disturbances are common and disabling in SCA3. Our previous studies have demonstrated that these symptoms correlate with genetic and structural changes in the brain [11, 12, 13], and are related to both disease severity and duration [14]. However, the stage‐specific patterns have rarely been systematically modeled across the full disease continuum. In addition, neuroinflammation is a key feature of many neurodegenerative disorders. Translocator protein (TSPO) PET imaging, a well‐validated modality to detect in vivo microglial activation, has revealed early neuroinflammatory changes in Alzheimer's disease (AD) [15], Parkinson's disease (PD) [16], and Amyotrophic Lateral Sclerosis (ALS) [17]. Although neuropathological studies have demonstrated glial activation in the pons of SCA3 patients and mouse model [18, 19], neuroinflammation as a dynamic and spatially distributed process has not been adequately characterized in vivo in this disease.

In this context, we conducted a prospective, cross‐sectional study integrating neuropsychological assessment, structural MRI, and TSPO‐PET imaging in a cohort of *ATXN3* mutation carriers across the full spectrum of disease stages. By evaluating non‐motor symptoms, neuroanatomical atrophy, and neuroinflammation across disease stages, we aim to map the multi‐modal biomarker patterns of SCA3.

## 2 Materials and Methods

### 2.1 Standard Protocol Approvals, Registrations and Patient Consent

The study was approved by the Ethics Committee for Medical Research of the First Affiliated Hospital of Fujian Medical University ([2019]195) and was conducted in accordance with the ethical guidelines and regulations of the committee and the Declaration of Helsinki. All participants gave written informed consent.

### 2.2 Study Participants

In this prospective, cross‐sectional cohort study, all participants were recruited consecutively from the Organization in South‐East China for Cerebellar Ataxia Research (OSCCAR) in the department of neurology of the First Affiliated Hospital of Fujian Medical University in China from February 2019 to February 2023. All patients had genetically confirmed SCA3 with CAG repeat expansion in *ATXN3* alleles ranging from 56 to 87 [20]. Sex‐ and age‐ matched healthy control individuals (HC) with normal CAG repeat numbers ranging from 12 to 44 [20] were recruited at the same hospital.

To ensure the accuracy and reliability of the findings, The following exclusion criteria were applied for all patients: (a) under 18 years old; (b) previous or current addiction to substances; (c) a history of other neurologic or systemic diseases; (d) impossibility to perform MRI and PET examinations for any reason (e.g., claustrophobia, or pacemaker); (e) radiation exposure would exceed the annual guidelines; (f) other causes of structural lesions on MRIs; and (g) for women, pregnancy, lactation, or lack of efficient contraception.

TSPO *rs6971* polymorphism was genotyped from peripheral blood DNA [21]. Among the 72 participants, 69 (95.8%) were high‐affinity binders and 3 (4.2%) were mixed‐affinity binders; no low‐affinity binders were identified. Consequently, no patients (0%) were excluded based on TSPO binding status, consistent with the approximately 10‐fold lower frequency of low‐affinity binders in East Asian populations compared with Caucasians [22].

Each participant included in the analysis underwent at least one assessment, including neuropsychological evaluation, brain MRI, or TSPO‐PET.

### 2.3 Neuropsychological Tests

Non‐motor symptoms were evaluated using a battery of standardized assessments. Detailed description of the neuropsychological assessments has been described in our previous reports [11, 13]. Cognitive function was screened using the Montreal Cognitive Assessment (MoCA), with scores ≤ 25 indicating cognitive impairment [14, 23]. Depression and anxiety symptoms were assessed using the Hamilton Depression Rating Scale (HAMD) [24, 25] and the Hamilton Anxiety Rating Scale (HAMA) [26], respectively. A HAMA score ≥ 8 was considered indicative of anxiety, and a HAMD score ≥ 8 was considered indicative of depression [27, 28]. Additionally, fatigue, sleep quality, and daytime sleepiness were evaluated using the Fatigue Scale‐14 (FS‐14) [29], Pittsburgh Sleep Quality Index (PSQI) [30], and the Epworth Sleepiness Scale (ESS) [31], respectively. Fatigue was defined as a FS‐14 score ≥ 7, poor sleep quality as a PSQI score ≥ 6, and excessive daytime sleepiness (EDS) as an ESS score ≥ 10 [30, 31, 32].

To ensure standardization and reliability of data, all neuropsychological tests were performed by one trained team (LLQ, XYC, and CYH), with participants tested in the same manner, during the day, in a quiet room, and in a fixed order.

### 2.4 Clinical Assessment

The presence and severity of ataxia in all participants were assessed using the Scale for the Assessment and Rating of Ataxia (SARA) [6]. Patients with a SARA score < 3 were defined as preataxic SCA3; those with SARA score ≥ 3 were defined as ataxic SCA3 [7]. “Age” refers to the age at examination for MRI, PET or neuropsychological tests. For the 38 pre‐manifest SCA3 mutation carriers without gait disturbances, the predicted age at onset was calculated based on CAG repeat length, adjusted for their age at baseline evaluation [33]. “Disease duration” denotes the interval from the date of the first symptom onset to the date of the examination for MRI, PET or neuropsychological tests, with negative values indicating predicted time to ataxia onset.

### 2.5 Image Acquisition

Participants underwent 3.0‐Tesla brain MRI examinations using a Siemens Skyra scanner (Siemens Healthcare, Erlangen, Germany; software version: syngo MR E11). Sagittal anatomical images were acquired using a 3‐dimensional T1‐weighted magnetization‐prepared rapid gradient‐echo (MP‐RAGE) sequence with the following scan parameters: repetition time = 2300 ms, echo time = 2.3 ms, inversion time = 900 ms, flip angle = 8°, field of view = 240 mm × 256 mm, number of slices = 192, voxel size = 1 × 1 × 1 mm^3^, total acquisition time = 5.2 min. After completing the MRI scan, all data sets were checked by one experienced radiologist for quality control, including motion correction and cutoffs for head movement; 5 SCA3 patients were excluded due to excessive motion artifacts.

^18^F‐DPA‐714 PET examinations were conducted using a hybrid Siemens Biograph mCT PET/CT system (Biograph mCT 64, Siemens Healthcare, Erlangen, Germany; software version: syngo VG60A). Images were acquired between 60 and 90 min after intravenously injecting a bolus of 10–15 mCi ^18^F‐DPA‐714. Following a low‐dose CT scan for attenuation correction, a PET scan was immediately performed. All the collected data were transferred to the Multimodality Workstation (syngo MI, SIEMENS, Germany). PET data were reconstructed to a final voxel size of 1 × 1 × 1 mm^3^, using the ordered subset expectation maximization algorithm (2 iterations and 21 subsets), and CT data were utilized for attenuation correction.

### 2.6 Image Postprocessing

Structural MRI brain volumetry was automatically performed using the vol2Brain module (version 1.0, release 23‐11‐2021) on the volBrain platform (currently hosted at https://volbrain.net/home) [34]. To ensure strict analytical reproducibility and prevent confounding effects from potential algorithmic updates, all MRI datasets were processed in a batched manner within a single, concentrated time in December 2024. Regional volumes of interest (ROIs) were automatically segmented and quantitatively extracted in MNI space (neurological orientation) using the native vol2Brain pipeline. The ROIs included: (1) brainstem; (2) cerebellar white matter (CWM); (3) cerebellar gray matter (CGM); (4) ventral diencephalon (Ventral DC); (5) pallidum; (6) putamen; (7) caudate; (8) thalamus. All ROI volumes were normalized by each participant's total intracranial volume (TIV). To generate representative MRI figures, native T1WI datasets were spatially normalized to the Montreal Neurological Institute 152 (MNI152) standard space using SPM12 (version r7771, https://www.fil.ion.ucl.ac.uk/spm/software/spm12) implemented in MATLAB R2022b (The MathWorks, Natick, MA, USA). Representative slices across groups were then extracted at identical MNI coordinates.

For PET scans, reconstructed dynamic data were realigned for motion correction according to the process of frame‐to‐reference image registration in Pmod software (version 4.0; PMOD Technologies Ltd.). The SUV maps of ^18^F‐DPA714 were calculated from the frames between 60 and 90 min of acquisition (SUV60‐90) as the radioactivity concentration divided by injected dose per body mass. SPM12 MNI atlas was used for ROIs extraction. Standard uptake value ratio (SUVR) was calculated for each ROI using the cerebellar cortex as the reference region [35, 36], as cerebellar cortical ^18^F‐DPA714 SUV remained stable across HC and SCA3 disease stages, with no significant group differences observed after adjusting for age and sex (*p* = 0.162; Figure S1).

### 2.7 Statistical Analysis

All data were analyzed using SPSS (version 26) and R Software (version 4.3.2). Continuous variables with normal distribution were presented as mean (SD). Non‐normally distributed data are presented as medians alongside their inter‐quartile range (IQR) and categorical variables as number (%). Normality was assessed using the Kolmogorov–Smirnov test. The Mann–Whitney *U* test was used for nonparametric continuous variables, while independent samples *t*‐test was applied for normally distributed variables. Categorical variables were analyzed using chi‐squared test (Fisher's exact test when expected values were < 5). Analysis of Covariance (ANCOVA) was used for evaluating the differences in cerebellar cortex SUV across HC and proposed SCA3 staging, entering age and sex as covariates. Comparisons of MRI‐derived volumes and ROI‐SUVR values between SCA3 patients and HC were conducted using multiple linear regression models, adjusting for age and sex. *p* values were corrected for multiple comparisons using the Benjamini‐Hochberg false discovery rate (FDR) method.

To investigate the associations of non‐motor symptom scores, MRI‐derived volumes and ROI‐SUVR values with disease duration, additive regression models were applied. Prior to analyses, MRI‐derived volumes and ROI‐SUVR values were age‐adjusted using z‐transformation to enable comparability across measures, as described in detail in the supporting information [37]. Since non‐motor symptoms have established diagnostic thresholds, raw scores were analyzed without transformation. Additive regression models were then fitted using cubic P‐splines with six B‐spline basis functions and a second‐order difference penalty (bs = “ps”, *k* = 6, = 2 in R package mgcv, version 1.9–3) [38].

Receiver operating curve (ROC) analyses were applied to estimate the discriminative ability of the MRI‐derived volumes and ROI‐SUVR values between SCA3 patients and HC. The R package “pROC” was used for the visualization, with the area under the curve (AUC) reported for each biomarker.

## 3 Results

### 3.1 Demographic and Clinical Characteristics

A total of 229 SCA3 patients (with CAG repeats ranging from 58 to 85) and 104 HC (with CAG repeats ranging from 13 to 43) were included in this study. Patients with SCA3 and HC were matched for age and sex (*p* > 0.05). Among the SCA3 patients, 38 (16.6%) were preataxic and 191 (83.4%) were ataxic. Detailed demographic and clinical characteristics are shown in Table 1. Neuropsychological assessments were available for 125 SCA3 patients (26 preataxic and 99 ataxic) and 21 HC (Table S1). Structural MRI scans were available for 208 SCA3 patients (31 preataxic and 177 ataxic) and 61 HC (Table S2). ^18^F‐DPA‐714 PET imaging was available for 50 SCA3 patients (10 preataxic and 40 ataxic) and 22 HC (Table S3). Across all three modalities, both ataxic SCA3 and the total SCA3 cohort were well‐matched with HC in age and sex (*p* > 0.05), whereas pre‐ataxic SCA3 were younger than their respective HC subsets (*p* < 0.001 for neuropsychological tests, *p* = 0.006 for MRI, and *p* = 0.002 for ^18^F‐DPA‐714 PET; Tables S1–S3). Overlap across all three modalities was observed in 42 SCA3 patients (9 preataxic and 33 ataxic). Furthermore, overlap between neuropsychological tests and MRI was present in 62 SCA3 patients (10 preataxic and 52 ataxic), between MRI and ^18^F‐DPA‐714 PET in 6 SCA3 patients (0 preataxic and 6 ataxic), and between neuropsychological tests and ^18^F‐DPA‐714 PET in 2 SCA3 patients (1 preataxic and 1 ataxic). No HC contributed to more than one modality (Figure S3, Table S4).

**TABLE 1:** Baseline demographic and clinical characteristics of the overall study cohort.

	HC	Preataxic SCA3	Ataxic SCA3	Total SCA3	*P* ^a^
Total *n*	104	38	191	229	
Female sex, *n* (%)	53 (51.0)	21 (55.3)	81 (42.4)	102 (44.5)	0.276
Age, years, mean (SD)	39.7 (13.3)	30.6 (7.5)	41.6 (10.4)	39.8 (10.8)	0.944
Expanded CAG repeats number, median (IQR)	NA	75.0 (72.0, 76.0)	75.0 (73.0, 77.0)	75.0 (73.0, 77.0)	NA
Disease duration, years, median (IQR)	NA	−5.0 (−6.0, −4.0)	7.0 (4.0, 10.0)	6.0 (2.0, 9.0)	NA
Age at onset, years, median (IQR)	NA	33.0 (30.0, 39.0)	35.0 (27.0, 41.0)	35.0 (28.0, 41.0)	NA
SARA score, points, median (IQR)	NA	0 (0, 0)	9.5 (6.5, 12.5)	8.5 (4.5, 12.0)	NA

*Note:* Normally distributed data are presented as mean (±SD); non‐normally distributed data are presented as median (IQR); age is evaluated using an independent *t*‐test; the Chi‐square test (*χ* ^2^) is used for gender.

Abbreviations: HC, healthy control; IQR, interquartile ranges; NA, not applicable; SARA, scale for the assessment and rating of ataxia; SCA3, spinocerebellar ataxias Type 3; SD, standard deviation.

^a^ HC versus Total SCA3.

### 3.2 Non‐Motor Symptoms

Compared to HC, patients with SCA3 demonstrated lower scores in the MoCA (*p* < 0.001), higher scores in HAMA (*p* = 0.032) and FS14 (*p* < 0.001). There were no differences for HAMD and PSQI values between SCA3 patients and HC (Figure S2A, Table 2).

**TABLE 2:** Neuropsychological test scores, MRI and ^18^F‐DPA‐714 PET features of analyzed study groups.

Characteristics	HC	Preataxic SCA3	Ataxic SCA3	Total SCA3	*P* ^a^
Neuropsychological tests^b^					
MoCA	29 (27, 30)	28 (26, 29)	24 (21, 26)	25 (22, 27)	**< 0.001**
HAMA	3 (0, 7)	3 (1, 5)	8 (3, 14)	6 (3, 13)	**0.032**
HAMD	4 (2, 7)	2 (1, 4)	7 (2, 13)	5 (2, 11)	0.154
FS14	4 (2, 4)	3 (1, 6)	9 (5, 11)	7 (4, 11)	**< 0.001**
PSQI	3 (2, 6)	3 (2, 5)	5 (2, 7)	5 (2, 7)	0.476
ESS	NA	4 (1, 7)	4 (1, 9)	4 (1, 8)	NA
MRI features^c^					
Total intracranial volume (cm^3^)	1365.10 (1290.20, 1501.15)	1397.75 (1268.03, 1500.37)	1376.12 (1241.08, 1443.86)	1378.72 (1256.98, 1452.92)	0.171
Brainstem volume (ratio of TIV × 10^2^)	1.45 (1.38, 1.50)	1.34 (1.26, 1.38)	1.11 (1.04, 1.19)	1.14 (1.06, 1.24)	**< 0.001**
CWM volume (ratio of TIV × 10^2^)	2.34 (2.25, 2.54)	2.14 (1.97, 2.34)	1.70 (1.51, 1.92)	1.78 (1.54, 2.00)	**< 0.001**
CGM volume (ratio of TIV × 10^2^)	7.66 (7.34, 8.10)	7.34 (7.09, 7.94)	7.18 (6.72, 7.56)	7.22 (6.77, 7.58)	**< 0.001**
Ventral DC volume (ratio of TIV × 10^3^)	7.05 (6.86, 7.30)	6.78 (6.30, 7.03)	5.93 (5.59, 6.19)	6.02 (5.67, 6.32)	**< 0.001**
Pallidum volume (ratio of TIV × 10^3^)	2.16 (2.04, 2.27)	1.98 (1.83, 2.20)	1.67 (1.56, 1.83)	1.72 (1.58, 1.88)	**< 0.001**
Putamen volume (ratio of TIV × 10^3^)	6.64 (6.23, 7.00)	6.69 (6.17, 6.99)	6.04 (5.61, 6.38)	6.09 (5.65, 6.50)	**< 0.001**
Caudate volume (ratio of TIV × 10^3^)	5.37 (5.02, 5.73)	5.60 (5.00, 5.70)	5.02 (4.66, 5.28)	5.05 (4.71, 5.39)	**0.002**
Thalamus volume (ratio of TIV × 10^3^)	9.17 (8.83, 9.55)	9.25 (8.79, 9.63)	8.42 (8.03, 8.91)	8.56 (8.08, 9.03)	**< 0.001**
^18^F‐DPA‐714 PET features^d^					
Brainstem	1.39 (1.30, 1.44)	2.12 (2.03, 2.28)	2.30 (2.19, 2.44)	2.27 (2.08, 2.37)	**< 0.001**
CWM	1.11 (1.07, 1.16)	1.07 (1.06, 1.09)	1.11 (1.07, 1.16)	1.09 (1.07, 1.15)	0.762
Ventral DC	1.33 (1.24, 1.39)	1.75 (1.67, 1.81)	1.78 (1.64, 1.91)	1.77 (1.66, 1.88)	**< 0.001**
Pallidum	1.11 (1.04, 1.14)	1.06 (1.01, 1.12)	1.24 (1.12, 1.41)	1.17 (1.08, 1.35)	**< 0.001**
Putamen	1.01 (0.98, 1.08)	0.91 (0.86, 0.92)	0.98 (0.93, 1.09)	0.95 (0.91, 1.04)	0.501
Caudate	0.82 (0.75, 0.87)	0.72 (0.64, 0.73)	0.69 (0.62, 0.81)	0.69 (0.63, 0.76)	**0.013**
Thalamus	1.28 (1.17, 1.32)	1.36 (1.29, 1.45)	1.42 (1.34, 1.50)	1.39 (1.33, 1.48)	**< 0.001**

*Note:* data are presented as median (IQR); Statistical differences are determined by multiple linear regression models, adjusting for age and sex. *p* values were corrected for multiple comparisons using the Benjamini‐Hochberg false discovery rate (FDR) method. The bold values indicate statistically significant results (*p* < 0.05). ^18^F‐DPA‐714 uptake of brain region is normalized according to the corresponding ^18^F‐DPA‐714 uptake in the cerebellar cortex.

Abbreviations: CGM, cerebellar gray matter; CWM, cerebellar wight matter; ESS, Epworth Sleeping Scale; FS‐14, Fatigue Scale‐14; HAMA, Hamilton Anxiety Scale; HAMD, Hamilton Depression Scale; HC, healthy control; MoCA, Montreal Cognitive Assessment; NA, not applicable; PSQI, Pittsburgh Sleep Quality Index; SARA, scale for the assessment and rating of ataxia; SCA3, spinocerebellar ataxias Type 3; Ventral DC, ventral diencephalon.

^a^ HC versus Total SCA3.

^b^ Neuropsychological tests were available in 21 HC and 125 SCA3 patients (26 preataxic SCA3 mutation carriers, 99 ataxic SCA3 mutation carriers).

^c^ MRI results were available in 61 HC and 208 SCA3 patients (31 preataxic SCA3 mutation carriers, 177 ataxic SCA3 mutation carriers).

^d^ ^18^F‐DPA‐714 PET results were available in 22 HC and 50 SCA3 patients (10 preataxic SCA3 mutation carriers, 40 ataxic SCA3 mutation carriers).

Using additive regression models, we investigated the relationships between non‐motor symptom scores and disease duration. We found that the overlap of MoCA, HAMA, HAMD, FS‐14 and PSQI scores with those of HC ended at 4.2 years (MoCA), 4.5 years (HAMA), 5.3 years (FS‐14), 6.3 years (HAMD), and 11.2 years (PSQI) after ataxia onset. In contrast, ESS score remained within the normal range during the entire disease course (Figure 1).

**FIGURE 1:**
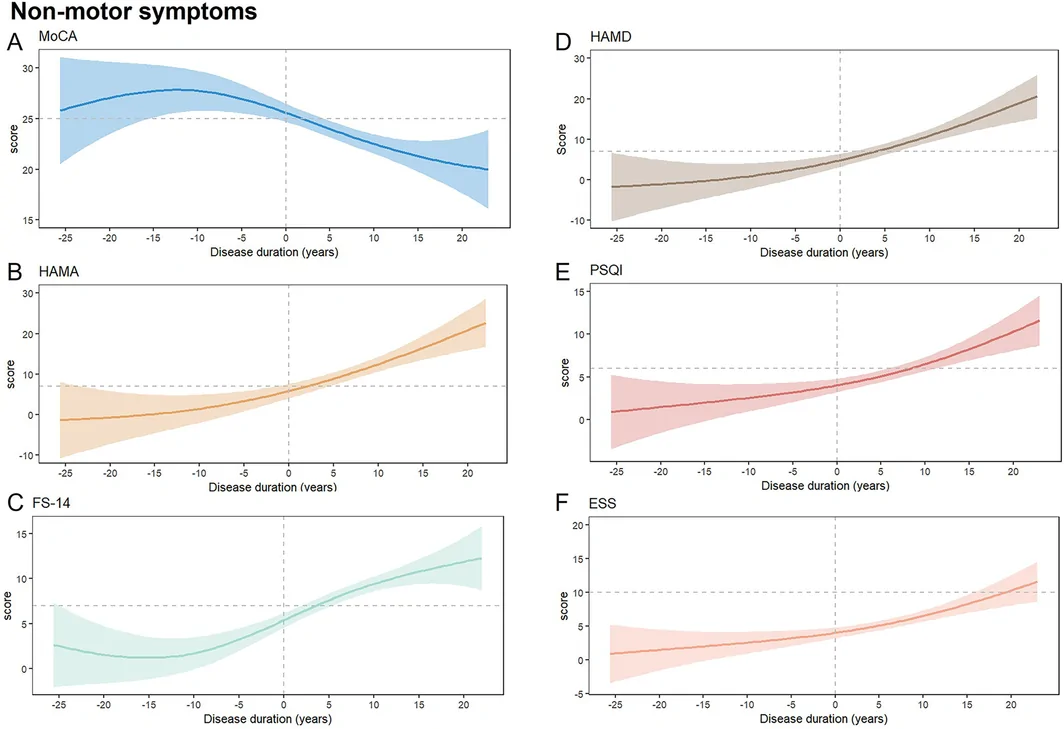
Scale for the assessment and rating of non‐motor symptoms scores in relation to disease duration. Data was analyzed with additive regression models on a time scale defined by disease duration. The estimated 95% confidence intervals are shown by the shaded areas around the curves. The time of ataxia onset is shown by a vertical dashed line in all graphs. (A) Montreal Cognitive Assessment (MoCA), with scores ≤ 25 indicating cognitive impairment. (B) Hamilton Anxiety Scale (HAMA), with scores ≥ 8 indicating anxiety. (C) Fatigue Scale‐14 (FS‐14), with scores ≥ 7 indicating fatigue. (D) Hamilton Depression Scale (HAMD), with scores ≥ 8 indicating depression. (E) Pittsburgh Sleep Quality Index (PSQI), with scores ≥ 6 indicating poor sleep quality. (F) Epworth Sleeping Scale (ESS), with scores ≥ 10 indicating daytime sleepiness. Cut‐off is given as a dashed horizontal line in graphs.

### 3.3 MRI Parameters

Compared with HC, patients with SCA3 demonstrated volume loss in brainstem (*p* < 0.001), CWM (*p* < 0.001), CGM (*p* < 0.001), ventral DC (*p* < 0.001), pallidum (*p* < 0.001), putamen (*p* < 0.001), caudate (*p* = 0.002), and thalamus (*p* < 0.001) (Figure S2B, Table 2).

Associations between regional volumes and disease duration in SCA3 are shown in Figure 2A and Figure S4A. The overlap of Brainstem and Ventral DC volumes with those of HC ended at 0.7 years and 0.2 years before ataxia onset, respectively. In contrast, the overlap of CWM and pallidum volumes with those of HC ended at 0.7 years and 4.8 years after ataxia onset. Volumes in CGM, putamen, caudate, and thalamus decreased slightly but remained within the ±2 SD of the control mean during the entire disease course.

**FIGURE 2:**
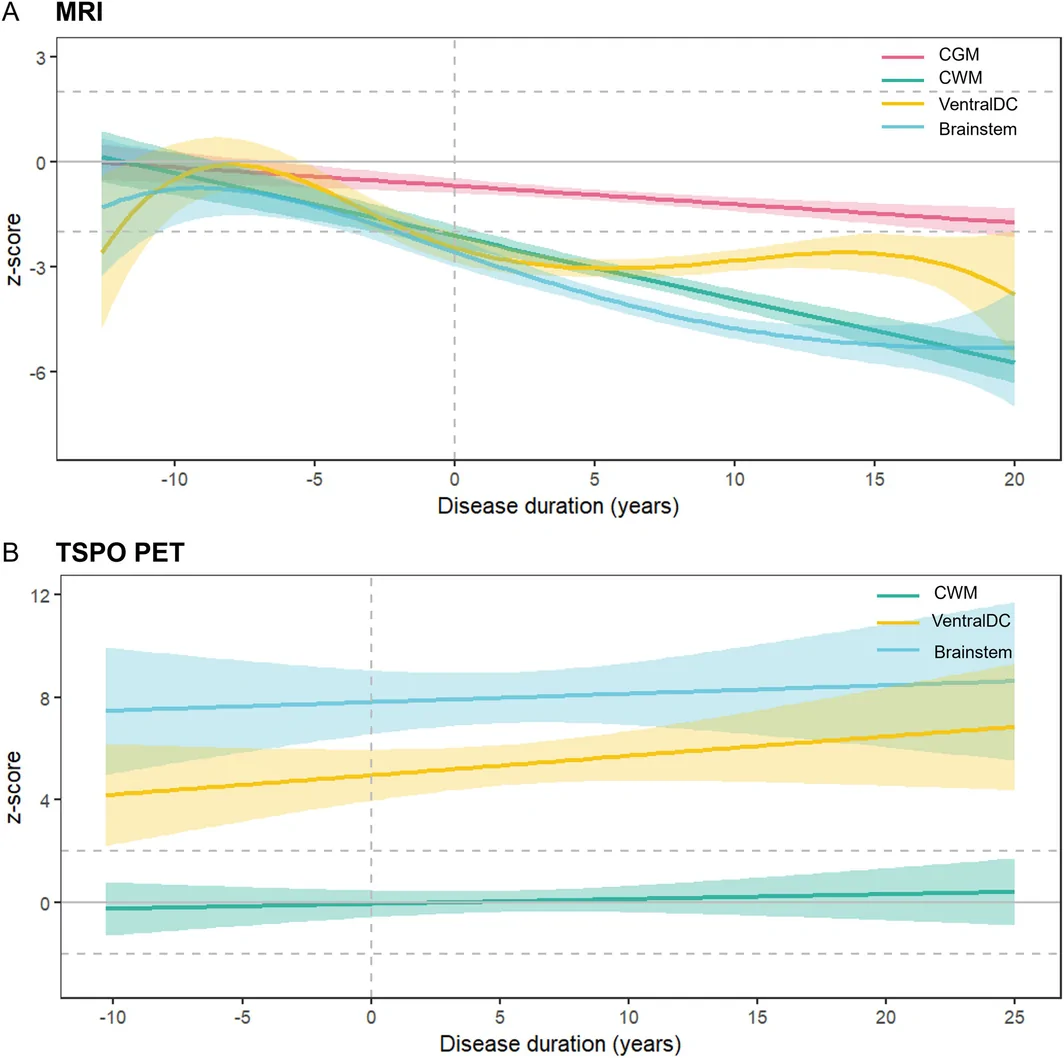
Imaging biomarkers in relation to disease duration. (A) Volumes of cerebellar wight matter (CWM), cerebellar gray matter (CGM), ventral diencephalon (ventral DC) and brainstem. (B) SUVR values of brainstem, CWM and ventral DC. Data were *z*‐transformed in relation to healthy controls of the same age and analyzed with additive regression models on a time scale defined by disease duration. The estimated 95% confidence intervals are shown by the shaded areas around the curves. The time of ataxia onset is shown by a vertical dashed line. The mean of healthy controls is given as a horizontal solid line, and the 2 SD range by dotted lines.

ROC curve analyses illustrated that the volume in brainstem and Ventral DC could accurately differentiate total SCA3 patients from HC (AUC = 0.9639 and 0.9284, respectively; Figure S5A), whereas their discriminative performance was lower for preataxic SCA3 and HC (AUC = 0.7885 and 0.6631, respectively; Figure S5B).

### 3.4 ^18^F‐DPA‐714 Uptake Parameters

Compared with HC, patients with SCA3 demonstrated higher ^18^F‐DPA‐714 uptake in brainstem (*p* < 0.001), ventral DC (*p* < 0.001), pallidum (*p* < 0.001), and thalamus (*p* < 0.001), whereas uptake was lower in the caudate (*p* = 0.013) after FDR correction (Figure S2C, Table 2).

Associations between ROI‐SUVR values and disease duration in SCA3 patients are shown in Figure 2B and Figure S4B. SUVR values in the brainstem and ventral DC of SCA3 were higher than those of HC throughout the disease course. In the pallidum, SUVR value increased and deviated from normal 10 years after ataxia onset. In contrast, SUVR value in the putamen, caudate, and thalamus remained within ±2 SD of the control mean during the entire disease course.

ROC curve analyses illustrated that the SUVR values in brainstem and Ventral DC could accurately differentiate total SCA3 patients from HC (AUC = 0.97 and 0.9582, respectively; Figure S5C), and also showed good discriminative performance for preataxic SCA3 and HC (AUC = 0.9 and 0.9273, respectively; Figure S5D).

### 3.5 Disease Stages Definition

Based on the temporal sequence of non‐motor symptom scores, MRI‐derived volumes and ROI‐SUVR values, we defined distinct stages of disease progression in SCA3. First, the TSPO‐PET biomarker stage includes preataxic SCA3 patients who show increased ^18^F‐DPA‐714 uptake in the brain, in the absence of overt regional brain atrophy (defined by a SARA score < 3 and a brainstem volume *z*‐score > −2). Second, the MRI biomarker stage is characterized by the presence of significant regional brain volume loss on structural MRI in SCA3 patients who remain clinically preataxic. This stage is defined by a SARA score < 3 and a brainstem volume *z*‐score ≤ −2. Third, the ataxia stage includes individuals meeting the clinical criteria for ataxia (SARA ≥ 3) while still preserving global cognition, defined as a MoCA score ≥ 26. MoCA was chosen as a criterion due to its sensitivity to early cognitive changes, as observed in previous analyses. Four, the final non‐motor symptom stage includes ataxic mutation carriers (SARA ≥ 3) who develop clinically significant non‐motor symptoms, reflected by a MoCA score < 26. These stage definitions represent a sequential model of SCA3 progression, beginning with early neuroinflammation, followed by neurodegeneration, motor dysfunction, and subsequent cognitive decline (Figure 3). Representative TSPO‐PET and MRI images across disease stages of SCA3 are shown in Figure 4.

**FIGURE 3:**
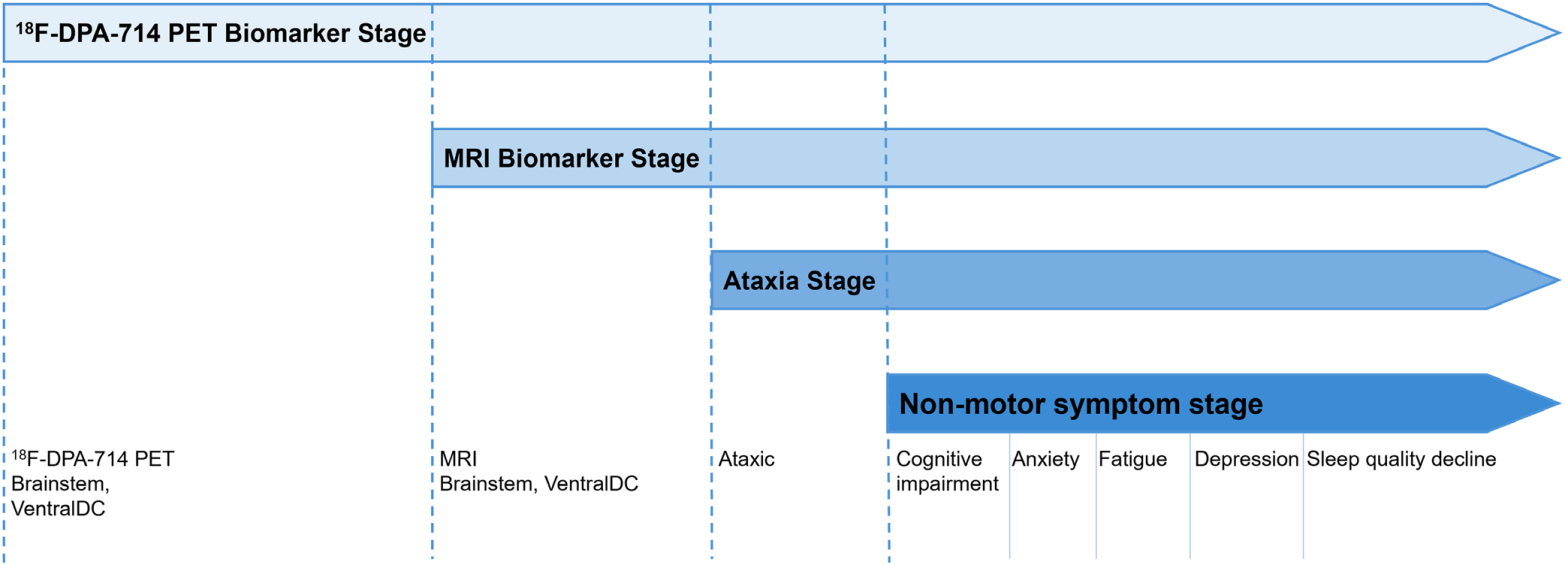
Staging model of spinocerebellar ataxia Type 3 (SCA3). Proposed staging model of SCA3 based on the temporal sequence of translocator protein positron emission tomography (TSPO‐PET) and MRI biomarker and symptoms changes. The model includes an initial TSPO PET biomarker stage, preataxic SCA3 have significant ^18^F‐DPA‐714 uptake increase in brainstem and Ventral DC in this stage. Followed by MRI biomarker stage defined by absence of ataxia, but with obvious regional volume loss also in brainstem and Ventral DC. Next is the ataxia stage, defined by manifest ataxia. The ataxia stage leads into the non‐motor symptom stage, in which cognitive functions, anxiety, fatigue, depression, and sleep quality declined after ataxia onset in SCA3. Ventral DC = ventral diencephalon.

**FIGURE 4:**
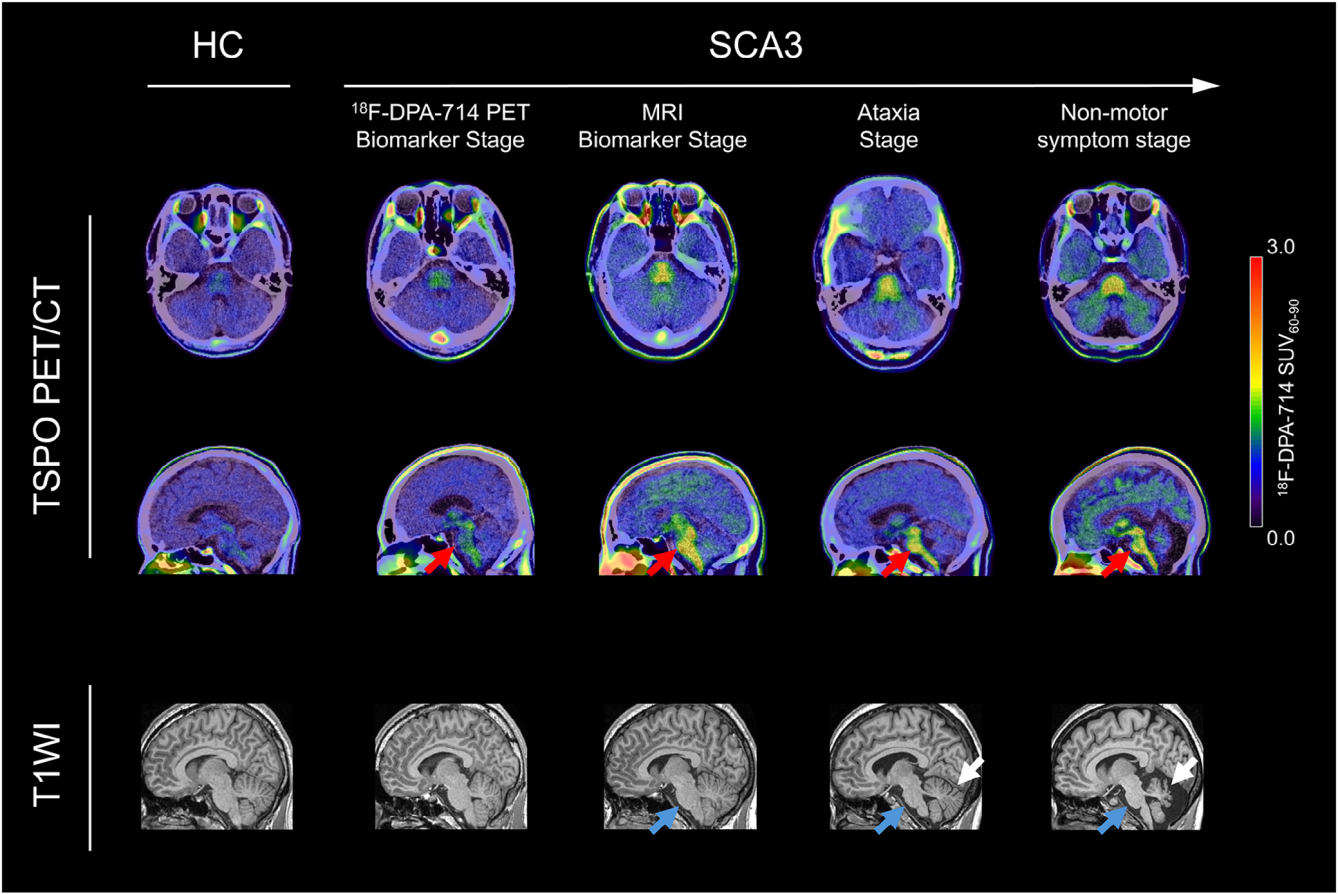
Representative ^18^F‐DPA‐714 PET and MRI images across the proposed spinocerebellar ataxia Type 3 (SCA3) staging. All representative structural T1‐weighted MR images are presented in MNI standard space at identical spatial slice coordinates. Except for the healthy control (HC) group, representative PET and MRI images within each column of SCA3 are obtained from the same individual subject. TSPO signal in the brainstem (red arrows) was increased and deviated from normal throughout the disease course. TSPO signal in brainstem (red arrow) was increased and deviated from normal throughout the disease course. Volume loss in the brainstem (blue arrows) occurred before ataxia onset, whereas volume loss in the cerebellum (white arrows) was observed after ataxia onset.

## 4 Discussion

In this prospective, cross‐sectional cohort study, we analyzed the data from 229 patients with SCA3 to characterize the temporal progression of non‐motor symptoms, MRI‐derived volumes and ROI‐SUVR values across the disease course. Based on these observations, we proposed a four‐stage model of SCA3 progression. The first stage, the ^18^F‐DPA‐714 PET biomarker stage, includes preataxic patients with increased ^18^F‐DPA‐714 uptake in the absence of structural brain changes. The second stage, the MRI biomarker stage, involves preataxic patients with regional volume loss on MRI. The third stage, the ataxia stage, is defined by the presence of manifest ataxia. Finally, the non‐motor symptoms stage encompasses ataxic patients who develop clinically non‐motor symptoms.

In SCA3, the presence of abnormally expanded ataxin‐3 protein and its toxic effect initiate the disease process and ultimately lead to irreversible neuronal degeneration throughout the brain [2, 39]. Neuroinflammatory activation is considered a response associated with abnormal protein accumulation, which contributes to disease progression, consistent with mechanisms observed in other neurodegenerative disorders [17, 40, 41, 42, 43, 44]. Previous studies in SCA3 mouse models have reported that reducing neuroinflammation significantly ameliorates neuropathological changes and improves motor coordination [45, 46]. In the present study, we used the ^18^F‐DPA‐714 PET approach to quantify brain inflammation in vivo, and found increased ^18^F‐DPA‐714 uptake in the brainstem and ventral DC at the early disease stage. The distribution of elevated tracer uptake paralleled regions of early structural vulnerability in SCA3 and emerged at least a decade before clinical onset, supporting the notion that neuroinflammation might play an active, early role in the pathogenic cascade rather than serving merely as a late‐stage byproduct of neurodegeneration.

A critical methodological consideration in TSPO‐PET quantification is the selection of an appropriate reference region. Neuropathological evidence in SCA3 reveals SCA3 often affect dentate nucleus, basal ganglia, substantia nigra, spinal cord and polyneuropathy [1], and structural MRI has revealed the volume loss in the cerebellar white matter, brainstem, and basal ganglia, with progressive worsening across disease stages, whereas the cerebellar cortex remains relatively spared, the CGM (cerebellar cortex) volume stayed within the 2 SD range around the mean of controls during the entire disease course [9, 38]. Given its high pathological and volumetric stability, the cerebellar cortex was selected as the pseudo‐reference region for SUVR calculations. To empirically confirm its baseline stability, we evaluated the SUV values of the cerebellar cortex across the stages (Figure S1). After controlling for potential demographic confounders using ANCOVA with age and sex as covariates, raw SUV values in the cerebellar cortex exhibited no significant differences across groups (*p* > 0.05). Thus, the progressive increase in brainstem ^18^F‐DPA‐714 PET metrics across biomarker stages reflects a conservative estimate of disease‐related neuroinflammation rather than a reference region artifact.

Both fluid NfL [47, 48] and ^18^F‐DPA‐714 PET neuroinflammation signals demonstrate pronounced abnormalities during the pre‐ataxic phase but exhibit relatively modest progression across subsequent manifest stages. This reflects the underlying biological nature of both indices as dynamic rate and response markers. Specifically, fluid NfL might reflect the ongoing axonal damage. In the pre‐ataxic stage, active stress and injury across a large pool of preserved yet affected axons trigger increased NfL shedding, which subsequently plateaus as the disease advances due to progressive axonal loss and a chronic steady state of degeneration. Similarly, ^18^F‐DPA‐714 PET might capture an active microglial response. Prior to phenoconversion, mutant ATXN3 aggregation and early neuronal distress signals rapidly trigger microglial activation and TSPO upregulation. Throughout subsequent manifest stages, this neuroinflammatory cascade establishes a sustained plateau driven by a dynamic equilibrium between chronic pro‐inflammatory signaling, microglial phenotypic adaptation or exhaustion, and parenchymal tissue loss, consistent with microglial state transitions described in other neurodegenerative diseases such as Alzheimer's disease [49]. Consequently, both markers are exceptionally sensitive for early stratification and target engagement during pre‐ataxic stages. Importantly, preclinical studies in animal models of neuroinflammation and neurodegeneration have demonstrated that ^18^F‐DPA‐714 uptake is reversible and decreases significantly following anti‐inflammatory or immunomodulatory interventions [50, 51], reinforcing its utility as a quantifiable pharmacodynamic endpoint for monitoring target engagement and treatment response in future disease‐modifying trials.

MRI assessment for structural changes in SCA3 revealed volume loss in the brainstem, cerebellum, ventral DC, and basal ganglia in our study. Notably, the observation that brainstem and ventral DC fell below the normal range shortly before ataxia onset. These findings are in line with previous studies that reported the structural damage across the brainstem, the cerebellum, and the other brain regions and followed a specific temporal model, where degeneration spreads from brainstem–cerebellar circuits to basal ganglia and cortical regions [9, 10, 38]. This suggests that quantitative measurements of brainstem and ventral DC volumes may serve as valuable biomarkers to stratify mutation carriers at risk of imminent clinical onset. Additionally, these structural changes overlapped spatially with regions showing elevated ^18^F‐DPA‐714 uptakes, suggesting a potential link between neuroinflammatory processes and early neurodegeneration. Importantly, the structural atrophy appeared to occur later in time than the ^18^F‐DPA‐714 signal elevation, implying that inflammatory activation may precede and possibly contribute to subsequent tissue volume loss in the preataxic stage of SCA3. Beyond conventional volumetry, advanced MR modalities provide critical sensitivity across the SCA3 spectrum. While structural T1 volumetry primarily captures macroscopic tissue loss close to phenoconversion, diffusion MRI and ^1^H‐MRS detect microstructural breakdown (e.g., cerebellar peduncle diffusivity) and neurochemical deficits (e.g., pontine myo‐inositol and tNAA/tCr) in pre‐ataxic carriers well before overt atrophy occurs [52]. Longitudinally, while infratentorial volumetry (particularly pons) robustly tracks progressive tissue loss, longitudinal changes in middle cerebellar peduncle microstructure and pontine metabolites correlate directly with clinical functional decline (SARA and FARS‐ADL) [53]. Integrating advanced MR sequences with conventional morphometry will thus refine pre‐ataxic staging and trial endpoints.

The manifestation and progression of ataxia are associated with irreversible brain degeneration [1], our data demonstrate that the clinical onset of ataxia after early neuroinflammation and MRI‐detectable structural atrophy, highlighting the gap between subclinical pathology and clinical manifestation. The final stage in our model is characterized by the appearance of clinically significant non‐motor symptoms. Although the timing of non‐motor symptom emergence in our model appears to diverge from prior cross‐sectional studies reporting isolated non‐motor features around phenoconversion [14], this distinction stems from different methodological paradigms. Rather than recording binary symptom presence, we applied additive regression models to determine when continuous scale scores systematically crossed validated clinical diagnostic thresholds. Therefore, the proposed “non‐motor symptom stage” does not imply the absolute absence of subclinical features earlier in the disease course, but rather denotes the estimated disease‐time window at which continuous symptom scores reach clinically overt, meaningful cutoff levels. Subtle non‐motor abnormalities may emerge earlier, including around or even prior to phenoconversion, with overt impairment developing progressively across manifest stages. These non‐motor manifestations co‐occurred with progressive volumetric loss in the basal ganglia and thalamus alongside sustained TSPO elevation, suggesting that domain‐specific non‐motor deficits may be mediated by progressive dysfunction across fronto‐striatal‐thalamic networks rather than primary cerebellar pathology alone. Importantly, these features significantly impact quality of life and caregiver burden, highlighting the need for their systematic monitoring and incorporation into clinical staging frameworks.

Previous attempts to stage SCA3 have primarily relied on serum concentrations of NfL, MRI‐based atrophy markers, or clinical criteria such as the presence of ataxia. While valuable, these approaches lack sensitivity to the earliest disease processes and do not capture the full spectrum of non‐motor involvement. Our model advances the field by integrating TSPO‐PET as a marker of early neuroinflammation, structural MRI as a marker of neurodegeneration, and clinical assessments of motor and non‐motor function. This multimodal approach parallels developments in AD research, where biomarker‐driven staging (the ATN model) has transformed both mechanistic understanding and trial design [54]. Similarly, in Huntington's disease, longitudinal imaging studies have delineated premanifest structural changes that predict clinical onset [55]. By adopting a comparable framework, our study positions SCA3 within the broader landscape of biomarker‐defined neurodegenerative disorders while also highlighting unique disease‐specific pathways such as early brainstem inflammation.

There are several limitations in our study. First, our study is cross‐sectional, and while we reconstruct temporal trajectories using additive regression models, definitive inferences about within‐individual progression require longitudinal validation. Second, ^18^F‐DPA‐714 PET possesses inherent biological and methodological constraints: TSPO is expressed not only by activated microglia but also by reactive astrocytes and endothelial cells, which limits cellular specificity; ^18^F‐DPA‐714 binding depends on the TSPO *rs6971* genetic polymorphism, excluding low‐affinity binders hampers recruitment efficiency in rare SCA3 cohorts, highlighting the utility of state‐of‐the‐art, polymorphism‐independent radiotracers for future studies (e.g., ^18^F‐LW223, the third‐generation TSPO PET radiotracer); Furthermore, evaluating TSPO‐PET via reference‐region SUVR rather than gold‐standard dynamic arterial sampling is inherently less robust. Third, our staging definitions relied on specific cut‐offs (e.g., SARA ≥ 3, MoCA < 26) that, while clinically grounded, may not capture the full spectrum of subtle motor and cognitive changes. Finally, our cohort was relatively homogeneous in terms of ethnicity and recruitment site, and replication in independent, multicenter cohorts will be necessary to ensure generalizability.

In conclusion, we propose a four‐stage model of SCA3 progression that integrates ^18^F‐DPA‐714 PET, MRI, and clinical markers. This framework captures a sequential trajectory beginning with early neuroinflammation, followed by structural brain atrophy, manifest ataxia, and late‐stage non‐motor symptom emergence. Our findings underscore the central role of neuroinflammation in the earliest phase of SCA3, highlight the value of multimodal biomarkers, and provide a foundation for stage‐specific therapeutic strategies. By bridging molecular pathology with clinical outcomes, this staging model represents a step toward precision medicine in SCA3.

## Author Contributions

Y.F., S.‐R.G., S.‐B.Y., W.‐J.C., and N.W. formulated and designed the study concept. C.L., Z.‐X.Y., X.‐Y.C., L.‐L.Q., Y.‐S.Q., W.L., Z.C., R.‐Y.Y., X.‐T.Y., C.‐Y.H., X.‐Y.C., M.‐T.L., and S.‐R.G. enrolled the patients and conducted clinical assessments and neuropsychological tests. C.L., Z.‐X.Y., J.‐P.H., and S.‐B.Y. conducted imaging data collection. X.‐Y.C., X.‐T.Y., and W.L. performed laboratory determination. C.L., Z.‐X.Y., X.‐Y.C., S.‐B.Y., S.‐R.G., and Y.F. analyzed the data and interpreted the results. All authors were involved in drafting/revising the manuscript.

## Funding

This work was supported by the grants U21A20360 (Y.F.), 82371349(Y.F.), 82230039 (N.W.), and 82371879 (S.‐R.G.) from the National Natural Science Foundation of China and the grant 2022ZQNZD005 (Y.F.) from the Major Scientific Research Program for Young and Middle‐aged Health Professionals of Fujian Province.

## Conflicts of Interest

The authors declare no conflicts of interest.

## Supporting information


**Figure S1:** Evaluation of ^18^F‐DPA‐714 SUV stability in the cerebellar cortex reference region. Individual scatter dot plots displaying standard uptake values (SUV) of the cerebellar cortex across HC and proposed SCA3 staging. Horizontal lines represent group means. Group comparison evaluated by ANCOVA adjusting for age and sex revealed no statistically significant differences across groups (*p* = 0.162), confirming the stability of the cerebellar cortex as a suitable pseudo‐reference region for SUVR calculations.
**Figure S2:** Individual distributions and group comparisons of multimodal metrics. Violin and box plots overlaid with individual data points showing the distributions across HC, preataxic SCA3, ataxic SCA3, and total SCA3 cohorts for: (A) neuropsychological scores (MoCA, HAMA, HAMD, FS‐14, PSQI, and ESS), (B) MRI‐derived brain volumes normalized to TIV, and (C) regional ^18^F‐DPA‐714 PET SUVR values. In box plots, horizontal lines indicate medians, boxes represent interquartile ranges. *p* values at the top of each panel represent statistical comparisons between HC and total SCA3, determined by multiple linear regression models adjusting for age and sex. *p* values were corrected for multiple comparisons using the Benjamini‐Hochberg false discovery rate (FDR) method.
**Figure S3:** Venn diagrams of data collection distribution and overlap across assessment modalities. Proportional Venn diagrams depicting the sample sizes and intersection of participants who underwent neuropsychological tests (blue), structural MRI (orange), and ^18^F‐DPA‐714 PET imaging (pink) across study groups: HC (*n* = 104), preataxic SCA3 (*n* = 38), ataxic SCA3 (*n* = 191), and total SCA3 (*n* = 229). Data within each section are presented as participant counts and group percentages, *n* (%).
**Figure S4:** Changes of regional volume and ^18^F‐DPA‐714 uptake in relation to disease duration. (A) Volumes of pallidum, putamen, caudate and thalamus. (B) SUVR values of pallidum, putamen, caudate and thalamus. Data were z‐transformed in relation to healthy controls of the same age and analyzed with additive regression models on a time scale defined by disease duration. The estimated 95% confidence intervals are shown by the shaded areas around the curves. The time of ataxia onset is shown by a vertical dashed line. The mean of healthy controls is given as a horizontal solid line, and the 2 SD range by dotted lines.
**Figure S5:** The receiver operating characteristic (ROC) curves of biomarkers. ROC curves of MRI biomarkers in differentiating between total spinocerebellar ataxia Type 3 (SCA3) and healthy controls (HC) (A), as well as between preataxic SCA3 and HC (B). ROC curves of ^18^F‐DPA‐714 PET biomarkers in differentiating between total SCA3 and HC (C) as well as between preataxic SCA3 and HC (D). Ventral DC = ventral diencephalon.
**Table S1:** Demographic and clinical characteristics of the neuropsychological assessment sub‐cohort.
**Table S2:** Demographic and clinical characteristics of the MRI sub‐cohort.
**Table S3:** Demographic and clinical characteristics of the 18F‐DPA‐714 PET sub‐cohort.
**Table S4:** Distribution and overlap of data collection across assessment modalities.

## Data Availability

The data that support the findings of this study are available from the corresponding author upon reasonable request.
