# Supplementary material for: Stage‐Specific Patterns of Imaging and Non‐Motor Biomarkers in Spinocerebellar Ataxia Type 3
Source: Ann Clin Transl Neurol. 2026 Oct 13:10.1002/acn3.70555. Online ahead of print. doi: 10.1002/acn3.70555 (PMC13660296; doi:10.1002/acn3.70555)
Supplement: Supplementary file 1 [file ACN3-9999-0-s001.docx]

**Supplementary materials**

**Stage-specific patterns of imaging and non-motor biomarkers in spinocerebellar ataxia type 3**

Chun Li, MSc^1,2†^, Zhi-Xian Ye, MD^1,3†^, Xuan-Yu Chen, MSc^1†^, Liang-Liang Qiu, MSc^1,2^, Zhi-li Chen, MD^1^, Yu-Sen Qiu, MD^1^, Wei Lin, MSc^1^, Ru-Ying Yuan, MSc^1^, Xin-Tong Yu, MSc^1^, Chun-Yu Huang, MSc^1,2^, Xin-Yuan Chen, MSc^4^, Min-Ting Lin, MSc^1,2^, Jian-Ping Hu, MD, PhD^5^, Wan-Jin Chen, MD, PhD^1,2^, Ning Wang, MD, PhD^1,2^, Shao-Bo Yao, MD, PhD^1,2*^, Shi-Rui Gan, MD^1,2*^, Ying Fu, MD, PhD^1,2^^*^

^*^**Corresponding author:**

Ying Fu, Department of Neurology and Institute of Neurology of First Affiliated Hospital, Institute of Neuroscience, and Fujian Key Laboratory of Molecular Neurology, Fujian Medical University, Fuzhou 350005, China. Tel: 086-591-87982772; E-mail: [fuying@fjmu.edu.cn](mailto:fuying@fjmu.edu.cn).

Supplementary methods. Detailed description of modality-specific z-transformation

Figure S1. Evaluation of ^18^F-DPA-714 SUV stability in the cerebellar cortex reference region.

Figure S2. Individual distributions and group comparisons of multimodal metrics.

Figure S3. Venn diagrams of data collection distribution and overlap across assessment modalities.

Figure S4. Changes of regional volume and ^18^F-DPA-714 uptake in relation to disease duration

Figure S5. The receiver operating characteristic (ROC) curves of biomarkers.

Table S1. Demographic and clinical characteristics of the neuropsychological assessment sub-cohort

Table S2. Demographic and clinical characteristics of the MRI sub-cohort

Table S3. Demographic and clinical characteristics of the ^18^F-DPA-714 PET sub-cohort

Table S4. Distribution and overlap of data collection across assessment modalities.

**Supplementary methods**

**Detailed description of modality-specific z-transformation**

To account for the non-overlapping nature of the control cohorts and to control for physiological age-related variance, we utilized a modality-specific, age-stratified z-score approach, Specifically, within each modality (MRI and TSPO-PET), participants were divided into age brackets. Individual z-scores were computed using the mean and standard deviation of the corresponding age-matched HC subgroup.

$$z-scores=\frac{\text{Metric}_{\text{individual}}-\text{Mean}_{\text{HC, age-matched}}}{\text{SD}_{\text{HC, age-matched}}}$$

**Supplementary Figures**

**Figure S1. Evaluation of ^18^F-DPA-714 SUV stability in the cerebellar cortex reference region.** Individual scatter dot plots displaying standard uptake values (SUV) of the cerebellar cortex across HC and proposed SCA3 staging. Horizontal lines represent group means. Group comparison evaluated by ANCOVA adjusting for age and sex revealed no statistically significant differences across groups (*P* = 0.162), confirming the stability of the cerebellar cortex as a suitable pseudo-reference region for SUVR calculations.

**Figure S2. Individual distributions and group comparisons of multimodal metrics.** Violin and box plots overlaid with individual data points showing the distributions across HC, preataxic SCA3, ataxic SCA3, and total SCA3 cohorts for: (A) neuropsychological scores (MoCA, HAMA, HAMD, FS-14, PSQI, and ESS), (B) MRI-derived brain volumes normalized to TIV, and (C) regional ^18^F-DPA-714 PET SUVR values. In box plots, horizontal lines indicate medians, boxes represent interquartile ranges. *P* values at the top of each panel represent statistical comparisons between HC and total SCA3, determined by multiple linear regression models adjusting for age and sex. *P* values were corrected for multiple comparisons using the Benjamini-Hochberg false discovery rate (FDR) method.

**Figure S3. Venn diagrams of data collection distribution and overlap across assessment modalities.** Proportional Venn diagrams depicting the sample sizes and intersection of participants who underwent neuropsychological tests (blue), structural MRI (orange), and ^18^F-DPA-714 PET imaging (pink) across study groups: HC (n=104), preataxic SCA3 (n=38), ataxic SCA3 (n=191), and total SCA3 (n=229). Data within each section are presented as participant counts and group percentages, n (%).

**Figure S4. Changes of regional volume and ^18^F-DPA-714 uptake in relation to disease duration**. **(A)** Volumes of pallidum, putamen, caudate and thalamus. **(B)** SUVR values of pallidum, putamen, caudate and thalamus. Data were z-transformed in relation to healthy controls of the same age and analyzed with additive regression models on a time scale defined by disease duration. The estimated 95% confidence intervals are shown by the shaded areas around the curves. The time of ataxia onset is shown by a vertical dashed line. The mean of healthy controls is given as a horizontal solid line, and the 2 SD range by dotted lines.

**Figure S5. The receiver operating characteristic (ROC) curves of biomarkers.** ROC curves of MRI biomarkers in differentiating between total spinocerebellar ataxia type 3 (SCA3) and healthy controls (HC) **(A)**, as well as between preataxic SCA3 and HC **(B)**. ROC curves of ^18^F-DPA-714 PET biomarkers in differentiating between total SCA3 and HC **(C)** as well as between preataxic SCA3 and HC **(D)**. Ventral DC = ventral diencephalon.

| Table S1. Demographic and clinical characteristics of the neuropsychological assessment sub-cohort | | | | | | | |  |
| --- | --- | --- | --- | --- | --- | --- | --- | --- |
|  | HC | Preataxic SCA3 | Ataxic SCA3 | Total SCA3 | *P*-value^†^ | *P*-value^‡^ | *P*-value^§^ |  |
| Total *n* | 21 | 26 | 99 | 125 |  |  |  |  |
| Female sex, n (%) | 9 (42.9) | 12 (46.2) | 36 (36.4) | 48 (38.4) | 0.821 | 0.577 | 0.699 |  |
| Age, years, mean (SD) | 38.9 (12.2) | 29.0 (5.6) | 43.0 (10.8) | 40.1 (11.5) | **<0.001** | 0.122 | 0.656 |  |
| Expanded CAG repeats number,  median (IQR) | NA | 75.5 (73.5, 77.0) | 75.0 (72.0, 77.0) | 75.0 (72.0, 77.0) | NA | NA | NA |  |
| Disease duration, years, median (IQR) | NA | -4.0 (-5.3, -3.6) | 6.0 (4.0, 10.0) | 5.0 (2.0, 8.0) | NA | NA | NA |  |
| Age at onset, years,  median (IQR) | NA | 32.5 (30.0, 39.0) | 36.0 (29.0, 44.0) | 36.0 (29.0, 41.5) | NA | NA | NA |  |
| SARA score, points,  median (IQR) | NA | 0 (0, 0) | 9.0 (6.0, 12.0) | 7.0 (3.0, 11.0) | NA | NA | NA |  |
| Note: Normally distributed data are presented as mean (± SD); non-normally distributed data are presented as median (IQR); age using an independent t-test; the Chi-square test (χ2) is used for gender. Abbreviations: HC, healthy control; SARA, scale for the assessment and rating of ataxia; SCA3, spinocerebellar ataxias type 3; SD, standard deviation; IQR, interquartile ranges; NA, not applicable. ^†^HC *vs.* Preataxic SCA3. ^‡^HC *vs.* Ataxic SCA3. ^§^HC *vs.* Total SCA3. | | | | | | | |  |
|  |  |  |  |  |  |  |  |  |
|  |  |  |  |  |  |  |  |  |
|  |  |  |  |  |  |  |  |  |
|  |  |  |  |  |  |  |  |  |
|  |  |  |  |  |  |  |  |  |

| Table S2. Demographic and clinical characteristics of the MRI sub-cohort | | | | | | | |  |
| --- | --- | --- | --- | --- | --- | --- | --- | --- |
|  | HC | Preataxic SCA3 | Ataxic SCA3 | Total SCA3 | *P*-value^†^ | *P*-value^‡^ | *P*-value^§^ |  |
| Total *n* | 61 | 31 | 177 | 208 |  |  |  |  |
| Female sex, n (%) | 33 (54.1) | 17 (54.8) | 76 (42.9) | 93 (44.7) | 0.946 | 0.131 | 0.196 |  |
| Age, years, mean (SD) | 38.0 (13.3) | 30.7 (7.8) | 41.2 (10.3) | 39.7 (10.6) | **0.006** | 0.051 | 0.31 |  |
| Expanded CAG repeats number,  median (IQR) | NA | 74.0 (72.0, 76.0) | 75.0 (73.0, 77.0) | 75.0 (73.0, 77.0) | NA | NA | NA |  |
| Disease duration, years, median (IQR) | NA | -5.0 (-6.0, -4.0) | 7.0 (4.0, 10.0) | 6.0 (3.0, 9.0) | NA | NA | NA |  |
| Age at onset, years,  median (IQR) | NA | 33.0 (30.0, 39.0) | 35.0 (26.5, 40.5) | 34.0 (28.0, 40.0) | NA | NA | NA |  |
| SARA score, points,  median (IQR) | NA | 0.0 (0.0, 0.0) | 9.5 (6.5, 12.5) | 9.0 (5.0, 12.0) | NA | NA | NA |  |
| Note: Normally distributed data are presented as mean (± SD); non-normally distributed data are presented as median (IQR); age using an independent t-test; the Chi-square test (χ2) is used for gender. Abbreviations: HC, healthy control; SARA, scale for the assessment and rating of ataxia; SCA3, spinocerebellar ataxias type 3; SD, standard deviation; IQR, interquartile ranges; NA, not applicable. ^†^HC *vs.* Preataxic SCA3. ^‡^HC *vs.* Ataxic SCA3. ^§^HC *vs.* Total SCA3. | | | | | | | |  |
|  |  |  |  |  |  |  |  |  |
|  |  |  |  |  |  |  |  |  |
|  |  |  |  |  |  |  |  |  |
|  |  |  |  |  |  |  |  |  |
|  |  |  |  |  |  |  |  |  |

| Table S3. Demographic and clinical characteristics of the ^18^F-DPA-714 PET sub-cohort | | | | | | | |  |
| --- | --- | --- | --- | --- | --- | --- | --- | --- |
|  | HC | Preataxic SCA3 | Ataxic SCA3 | Total SCA3 | *P*-value^†^ | *P*-value^‡^ | *P*-value^§^ |  |
| Total *n* | 22 | 10 | 40 | 50 |  |  |  |  |
| Female sex, n (%) | 11 (50.0) | 3 (30.0) | 16 (40.0) | 19 (38.0) | 0.391 | 0.447 | 0.341 |  |
| Age, years, mean (SD) | 45.0 (13.6) | 29.9 (6.5) | 42.7 (9.3) | 40.1 (10.1) | **0.002** | 0.419 | 0.092 |  |
| Expanded CAG repeats number,  median (IQR) | NA | 75.0 (72.0, 76.0) | 74.0 (72.0, 75.0) | 74.0 (72.0, 75.0) | NA | NA | NA |  |
| Disease duration, years, median (IQR) | NA | -4.5 (-6.0, -3.0) | 7.0 (4.0, 10.5) | 5.5 (2.0, 8.0) | NA | NA | NA |  |
| Age at onset, years,  median (IQR) | NA | 33.0 (30.0, 39.0) | 35.5 (29.0, 41.0) | 34.5 (29.0, 41.0) | NA | NA | NA |  |
| SARA score, points,  median (IQR) | NA | 0.0 (0.0, 0.0) | 10.0 (6.0, 13.0) | 8.0 (3.0, 13.0) | NA | NA | NA |  |
| Note: Normally distributed data are presented as mean (± SD); non-normally distributed data are presented as median (IQR); age using an independent t-test; the Chi-square test (χ2) is used for gender. Abbreviations: HC, healthy control; SARA, scale for the assessment and rating of ataxia; SCA3, spinocerebellar ataxias type 3; SD, standard deviation; IQR, interquartile ranges; NA, not applicable. ^†^HC *vs.* Preataxic SCA3. ^‡^HC *vs.* Ataxic SCA3. ^§^HC *vs.* Total SCA3. | | | | | | | |  |
|  |  |  |  |  |  |  |  |  |
|  |  |  |  |  |  |  |  |  |
|  |  |  |  |  |  |  |  |  |
|  |  |  |  |  |  |  |  |  |
|  |  |  |  |  |  |  |  |  |

| Table S4. Distribution and overlap of data collection across assessment modalities. | | | | |  |
| --- | --- | --- | --- | --- | --- |
| Data Collection Overlap | HC | Preataxic SCA3 | Ataxic SCA3 | Total SCA3 |  |
| **All 3 Modalities** |  |  |  |  |  |
| Neuropsychology + MRI + PET | 0 (0%) | 9 (24%) | 33 (17%) | 42 (18%) |  |
| **Any 2 Modalities** |  |  |  |  |  |
| Neuropsychology + MRI only | 0 (0%) | 10 (26%) | 52 (27%) | 62 (27%) |  |
| MRI + PET only | 0 (0%) | 0 (0%) | 6 (3%) | 6 (3%) |  |
| Neuropsychology + PET only | 0 (0%) | 1 (3%) | 1 (1%) | 2 (1%) |  |
| **Only 1 Modality** |  |  |  |  |  |
| Neuropsychology only | 21 (20%) | 6 (16%) | 13 (7%) | 19 (8%) |  |
| MRI only | 61 (59%) | 12 (32%) | 86 (45%) | 98 (43%) |  |
| PET only | 22 (21%) | 0 (0%) | 0 (0%) | 0 (0%) |  |
| **Total Enrolled** | 104 (100%) | 38 (100%) | 191 (100%) | 229 (100%) |  |
| Note: Data are presented as n (%). In this study, PET specifically refers to ^18^F-DPA-714 PET Abbreviations: HC, healthy control; SCA3, spinocerebellar ataxias type 3. | | | | |  |
|  |  |  |  |  |  |
